# Foreign Body-induced Pancreatitis—Multimodal Imaging and Multispecialty Collaboration: A Case Report

**DOI:** 10.5811/cpcem.52940

**Published:** 2026-04-07

**Authors:** Nital Vaghela, Mohamad K. Abou Chaar, Steven C. Mahnke, Ceylan Colak, Daniel Stephens, Tobias Kummer

**Affiliations:** * Mayo Clinic, Division of Trauma, Critical Care and General Surgery, Rochester, Minnesota; † Mayo Clinic, Department of Emergency Medicine, Rochester, Minnesota.; ‡ Mayo Clinic, Department of Radiology, Rochester, Minnesota

**Keywords:** foreign body, pancreatitis, surgery, case report

## Abstract

**Introduction:**

Foreign body-induced pancreatitis is rare and diagnostically challenging, often presenting with non-specific symptoms and no clear history, unlike typical causes.

**Case Report:**

A 70-year-old man presented with vomiting and abdominal tenderness. Imaging revealed a 4-cm sharp foreign body near the pancreatic head causing inflammation. Endoscopy and endoscopic ultrasound failed to locate the object. Surgical exploration with intraoperative ultrasound identified and removed the foreign body at the pylorus-duodenal junction. The patient recovered without complications.

**Conclusion:**

Early diagnosis, multimodal imaging, and surgical collaboration are essential for optimal management of foreign body-induced pancreatitis.

## INTRODUCTION

Foreign body-induced pancreatitis is a rare cause of acute pancreatitis, with few cases described in the literature.^1^–^5^ Sharp objects like toothpicks or fish bones can perforate the gastrointestinal wall, and in about 1% of the cases they migrate to the pancreas or liver, causing inflammation that mimics acute pancreatitis.^6^–^8^ Diagnosis is frequently delayed due to the non-specific clinical presentation and the inability of patients to recall foreign body ingestion, highlighting the importance of early multimodal imaging and multidisciplinary evaluation.^9^,^10^ This case of a patient with foreign body-induced pancreatitis further demonstrates the diagnostic value of advanced imaging modalities and the integral role of collaborative management in guiding appropriate therapeutic intervention.^1^,^2^

## CASE REPORT

A 70-year-old male presented to the emergency department with vomiting, syncope, and abdominal pain that started two days earlier, associated with tenderness localized to the right upper quadrant and epigastric region. His medical history was notable for chronic hepatitis B without cirrhosis and recurrent episodes of pancreatitis in 2021 and 2022, with no identifiable cause. On physical examination, tenderness was noted in these areas. Vital signs were as follows: temperature, 37.8 °C; heart rate, 98 beats per minute; respiratory rate, 18 breaths per minute; blood pressure 151/73 mmHg; and oxygen saturation, 97%.

**Image 1:**
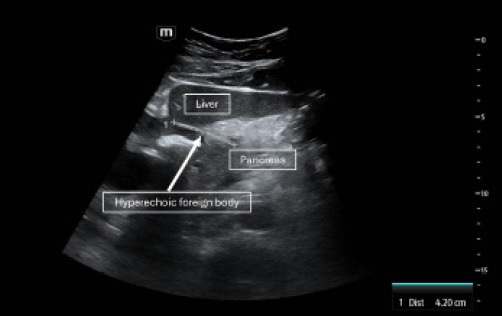
Gray-scale transverse routine point-of-care ultrasound image of the pancreas showing the foreign body (dotted line/arrow) in a patient diagnosed with foreign body-induced pancreatitis.

Laboratory investigations revealed a complete blood count showing hemoglobin at 10.6 grams per deciliter (g/dL) (reference range: 13.5–17.5 g/dL); hematocrit 34.1% (41–53%); leukocyte count 23.7 x10^3/microliter (μL) (4.0–11.0 ×10^3^/μL) with neutrophils 19.91 x10^3/μL (1.5–8.0 ×10^3^/μL). Basic metabolic panel results included sodium 133 millimoles per liter (mmol/L) (135–145 mmol/L); bicarbonate 19 mmol/L (22–29 mmol/L), serum calcium 8.4 milligrams per deciliter (mg/dL) (8.5–10.5 mg/dL); glucose 213 mg/dL (70–99 mg/dL (fasting) or < 140 mg/dL (random); and creatinine 1.62 mg/dL (0.6–1.3 mg/dL), with other electrolytes within normal limits. Liver function tests showed alanine aminotransferase 27 units per liter (U/L) (7–56 U/L); aspartate aminotransferase 21 U/L (10–40 U/L); alkaline phosphatase 118 U/L (40–129 U/); and serum lipase was 91 U/L (0–160 U/L). Given the right upper quadrant pain, a point-of care ultrasound (POCUS) was performed, which showed an unremarkable gallbladder but a 4.2-cm foreign body in close proximity to the pancreatic head with peripancreatic edema (Image 1, Video).

**Image 2:**
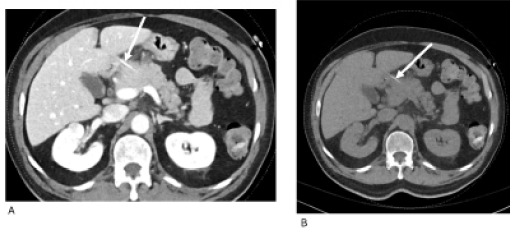
Axial computed tomography, with contrast (A), and without contrast (B), showing hyperdense linear foreign body (arrow) centered between the pancreatic head and antrum/duodenal region with adjacent inflammation.

A contrast-enhanced computed tomography (CT) of the abdomen confirmed the sharp foreign body with surrounding inflammatory changes near the pancreatic head and the antroduodenal region suspicious for foreign body-induced focal pancreatitis (Image 2).

**Image 3:**
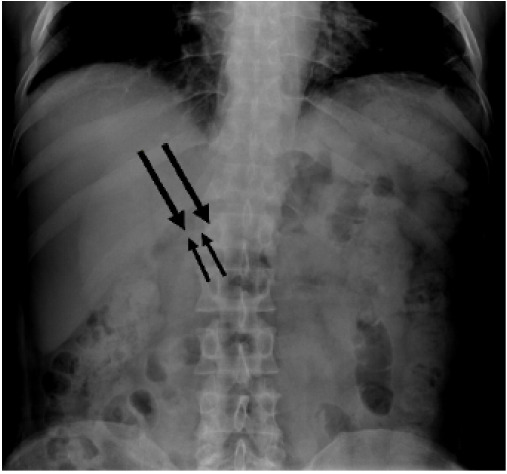
Anterior-posterior abdominal radiograph showing the foreign body (arrows).

The patient denied any history of ingesting foreign objects. Shortly after admission, he developed fever and neutrophilia and was started on broad-spectrum antibiotics (piperacillin-tazobactam). Pain was effectively managed with oral oxycodone and acetaminophen as needed. An esophagogastroduodenoscopy performed the following day was unremarkable, with no foreign body visualized. The patient was kept nil per os with intravenous fluids maintained for potential surgical intervention. A subsequent abdominal radiograph confirmed the persistence of the foreign body consistent with the initial ultrasound and CT findings (Image 3).

**Image 4:**
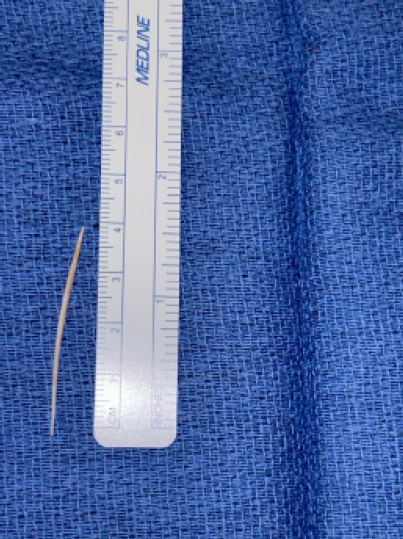
Intra-operative image showing the wooden foreign body next to a measurement tool.

Laboratory tests showed continued elevated lipase (91 U/L). Endoscopic ultrasound was attempted but failed to identify or retrieve the foreign object. After multidisciplinary discussion and informed consent, the patient was scheduled for open abdominal exploration to remove the foreign body. Intraoperatively, ultrasound was used to localize the foreign object near the pancreas but initially failed to detect it. Further dissection revealed severe tissue edema and induration around the gastrohepatic ligament. Repeat intraoperative ultrasound identified the foreign body anteriorly at the junction of the pylorus and the first portion of the duodenum, which was successfully removed (Image 4).

*CPC-EM Capsule*

What do we already know about this clinical entity?

*Foreign body-induced pancreatitis is rare, often mimics other causes, and is hard to diagnose without clear ingestion history*.

What makes this presentation of disease reportable?

*The use of multimodal imaging and surgical intervention after endoscopic failure highlights challenges that can be encountered in diagnosis and management*.

What is the major learning point?

*Early suspicion and multimodal imaging, including point-of-care ultrasound (POCUS), combined with multidisciplinary collaboration are key*.

How might this improve emergency medicine practice?

*Recognizing foreign body-induced pancreatitis and using POCUS early can expedite diagnosis and guide timely multidisciplinary management*.

The postoperative course was uneventful, and the patient recovered well, eventually being discharged in stable condition.

## DISCUSSION

Foreign body-induced pancreatitis is rare and diagnostically challenging, often presenting without a clear history of ingestion and with symptoms that mimic other causes of acute pancreatitis.^1^ While CT is consistently described as the key diagnostic modality in foreign body-induced pancreatitis,^11^ the combination of ultrasound, CT, endoscopy, endoscopic ultrasound, and intraoperative ultrasound was essential in our patient’s care. While both POCUS and CT identified the foreign body, both endoscopy and endoscopic ultrasound failed to retrieve it, highlighting the limitations of any single imaging modality in complex cases (Table). This case demonstrates that POCUS, a widely accessible tool in emergency and acute care settings, is a feasible adjunctive modality for raising early suspicion of pancreatic pathology. Due to the fixed position of the foreign body in our case, endoscopic retrieval was not feasible, which aligns with published literature that success rates decline when foreign bodies are fixed, extra-luminal, or associated with marked inflammatory changes.^8^,^10^

Surgical exploration remains the definitive treatment when less invasive methods fail. The literature, although limited to case reports and small series, consistently supports surgical intervention as necessary for safe foreign body removal, especially in the context of complications or persistent symptoms. Intraoperative ultrasound, as used here, can facilitate precise localization and minimize trauma to surrounding tissues.^10^ Multidisciplinary collaboration involving emergency medicine, radiology, gastroenterology, and surgery is critical for timely diagnosis and optimal management. Our patient’s favorable outcome reflects the value of teamwork and the integration of multiple imaging and therapeutic modalities.^12^

## CONCLUSION

Foreign body-induced pancreatitis is a rare and diagnostically challenging condition that often mimics other causes of acute pancreatitis. This case highlights the crucial role of multimodal imaging and multidisciplinary collaboration in achieving accurate diagnosis and effective management. Surgical exploration remains essential when less invasive methods fail, underscoring the importance of early recognition and a team-based approach to optimize patient outcomes.

## Supplementary Information

Video Point-of-care ultrasound demonstrating a linear echogenic structure consistent with a foreign body in the pancreas on a gray-scale transverse image.

## Figures and Tables

**Table:** Description of advanced imaging modalities, and their respective findings, in case of foreign-body-induced pancreatitis.

Imaging Modality	Findings
Point-of care ultrasound	4.2-cm foreign body near the pancreatic head with peripancreatic edema.
Contrast-enhanced abdominal computed tomography	Approximate 4-cm linear structure slightly abuts the superior pancreatic body and partially abuts/along the mildly thickened submucosa of the duodenal bulb/distal antrum, where there is mild adjacent stranding but without drainable fluid collection or overt pneumoperitoneum.
Esophagogastroduodenoscopy - endosonography	Localized moderate inflammation characterized by edema, erosion and erythema was found on the medial wall of the duodenal bulb. There was no foreign body noted intraluminally.
Abdominal radiograph	Thin linear radiopaque object projecting over the right paramedian upper abdomen at the level of the first lumbar vertebral body.
Endoscopic ultrasound	No discrete foreign object could be identified in the pancreatic head.
Intraoperative ultrasound	Foreign body lying anteriorly at the junction of the pylorus and first portion of the duodenum.
